# Genome-wide analysis of *basic helix–loop–helix* genes in *Dendrobium catenatum* and functional characterization of *DcMYC2* in jasmonate-mediated immunity to *Sclerotium delphinii*

**DOI:** 10.3389/fpls.2022.956210

**Published:** 2022-08-02

**Authors:** Cong Li, Xiang Cai, Qiuyi Shen, Xueliang Chen, Mengxi Xu, Tianqi Ye, Dun Si, Lingshang Wu, Donghong Chen, Zhigang Han, Jinping Si

**Affiliations:** State Key Laboratory of Subtropical Silviculture, National Forestry and Grassland Innovation Alliance on Dendrobium catenatum, SFGA Engineering Research Center for Dendrobium catenatum, Zhejiang A&F University, Hangzhou, China

**Keywords:** *Dendrobium catenatum*, jasmonate, *Sclerotium delphinii*, bHLH transcription factor, *DcMYC2b*

## Abstract

*Dendrobium catenatum*, belonging to the Orchidaceae, is a precious Chinese herbal medicine. *Sclerotium delphinii* (P1) is a broad-spectrum fungal disease, which causes widespread loss in the near-wild cultivation of *D. catenatum*. Thus, resistance breeding of *D. catenatum* has become the key to solve this problem. The *basic helix–loop–helix* (*bHLH*) gene family is closely related to plant resistance to external stresses, but the related research in *D. catenatum* is not deep enough yet. Phylogenetic analysis showed that 108 *DcbHLH* genes could be divided into 23 subgroups. Promoter cis-acting elements revealed that *DcbHLHs* contain a large number of stress-related cis-acting elements. Transcriptome analysis of MeJA and P1 treatment manifested that exogenous MeJA can change the expression pattern of most *bHLH* genes, especially the IIIe subgroup, including inhibiting the expression of *DcbHLH026* (*MYC2a*) and promoting the expression of *DcbHLH027* (*MYC2b*). Subcellular localization indicated that they were located in the nucleus. Furthermore, exogenous MeJA treatment significantly delayed disease time and reduced lesion size after infection with P1. *DcMYC2b*-overexpression Arabidopsis lines showed significantly smaller lesions after being infected with P1 than the wild type, indicating that *DcMYC2b* functions as an important positive regulator in *D. catenatum* defense against P1. Our findings shed more insights into the critical role of the *DcbHLH* family in plants and the resistance breeding of *D. catenatum*.

## Introduction

*Dendrobium catenatum* (*D. officinale*), a perennial herb of the genus *Dendrobium* in the Orchidaceae family, is a valuable traditional Chinese medicinal herb used for relieving upset stomach, promoting body fluid production, enhancing immunity, nourishing “yin,” and exerting antioxidant and anti-tumor effects (Chen et al., 2020b). In the past, *D. catenatum* has undergone a cross from natural wild to facility cultivation followed by near-wild cultivation (Chen et al., 2020b). Although the renewal of cultivation patterns has brought about species conservation and the rise of industry, resulting in great ecological and economic benefits (Si et al., 2017), it is susceptible to diseases in its large-scale cultivation, particularly southern blight disease, which causes substantial economic loss (Chen et al., 2019a). Southern blight disease is a broad-spectrum disease caused by the necrotrophic pathogen *Sclerotium delphinii* (P1) and *Sclerotium rolfsii* (Iquebal et al., 2017; Uji et al., 2017), and the former is more pathogenic (Uji et al., 2017). In warm and high humidity environments, *S. delphinii* infects the host and secretes numerous cell wall-degrading enzymes (CWDEs), leading to the degradation of the host cell wall (Li et al., 2017), followed by hyphal penetration of plant tissues, causing local necrosis and the formation of massive white mycelium and rapeseed-like sclerotia (Uji et al., 2017).

The family comprising basic helix–loop–helix (bHLH) transcription factors (TFs) is the second-largest family in eukaryotes (Ledent and Vervoort, 2001; Toledo-Ortiz et al., 2003), with a highly conserved bHLH domain, which can be divided into two functional regions: the basic region and the HLH region (Li et al., 2006). The former is located at the N-terminal end of the bHLH domain and associated with DNA binding, which enables the recognition of an E-Box (5′-CANNTG-3′) or G-box (5′-CACGTG-3′) upstream of a specific DNA sequence for the expression of genes involved in physiological and developmental processes and stress responses (Wang et al., 2015). The C-terminal HLH region is characterized by two amphipathic α-helices with a linking loop and is responsible for dimerization and interactions with other bHLH proteins (Heim et al., 2003). With the release of a vast number of genome sequences, more and more *bHLH* genes have been identified (Heim et al., 2003; Li et al., 2006, 2019; Yang et al., 2020; Zhou et al., 2020). In plants, the classification of bHLH proteins is usually based on the sequence homology of bHLH domains (Buck and Atchley, 2003; Pires and Dolan, 2010), with the number of subgroups ranging from 15 to 26. Some atypical bHLH proteins are even classified into 32 subgroups after phylogenetic analysis (Carretero-Paulet et al., 2010). There is a special subgroup within the family of bHLH TFs called myelocytomatosis oncogenes (MYC). Members of this subgroup contain a bHLH_MYC_N domain at their N-terminal end in addition to a bHLH domain at their C-terminal end (Chen et al., 2019b). Among them, the bHLH_MYC_N domain contains a JAZ interaction domain (JID) and a putative transcriptional activation domain (TAD). Previous studies have demonstrated that the family of bHLH TFs plays important role in plant growth and development (Hao et al., 2021). For example, 77 bHLH proteins in *Gossypium hirsutum* may be involved in brassinosteroid (BR) hormone signaling pathways to promote cotton fiber cell elongation (Lu et al., 2018). In Arabidopsis, MADS-Box and bHLH TFs coordinate transmitting-tract development, as well as bHLH IIIe subfamily member AtMYC2 functions synergistically with AtMYC3 and AtMYC4 in regulating leaf senescence (Di Marzo et al., 2020). Meanwhile, there are many *bHLH* genes involved in plant responses to biotic and abiotic stresses (Hao et al., 2021). *MdbHLH130*, an apple bHLH TFs, can improve plant tolerance to water deficit stress (Zhao et al., 2020). Overexpression of *Vitis amurensis VabHLH1* and *Vitis vinifera VvbHLH1* in Arabidopsis enhanced tolerance to low-temperature stress (Xu et al., 2014). In Arabidopsis, four IVc bHLH TFs (*bHLH34*, *bHLH104*, *bHLH105*/*ILR3-IAA-LEUCINE RESISTANT3*, and *bHLH115*) formed homodimers and heterodimers to regulate the Fe deficiency response and the Fe homeostasis (Zhang et al., 2015; Li et al., 2016; Liang et al., 2017; Tissot et al., 2019). Arabidopsis *bhlh104* mutant was sensitive to cadmium (Cd) stress, whereas plants overexpressing *bHLH104* exhibited enhanced Cd tolerance (Yao et al., 2018). The jasmonate (JA) signaling pathway primarily responds to plant immunity to necrotrophic fungal pathogens and herbivorous insects in the plant (Yan and Xie, 2015). AtMYC2, one of the core TFs of the JA signaling pathway, plays a crucial step in plant responses to biotic stresses (Dombrecht et al., 2007). For instance, AtMYC2, AtMYC3, and AtMYC4 act to decrease the feeding of *Spodoptera littoralis* by regulating glucosinolate biosynthesis (Schweizer et al., 2013). Moreover, AtMYC2 can directly bind to the promoters of terpenoid synthesis genes (*TPS10*, *TPS11*, and *TPS21*) and activate their expression to promote terpenoid synthesis, thereby enhancing terpenoid-mediated direct and indirect plant resistance to pests (Fernandez-Calvo et al., 2011; Adams et al., 2021). In *Oryza sativa*, the *OsMYC2*-RNAi lines showed enhanced resistance against bacterial pathogen *Xanthomonas oryzae pv. oryzae* (Giri et al., 2017). In tomatoes, SlMYC2 positively regulates both wounding-responsive genes by activating JA2L and pathogen-responsive genes by activating ERF.C3 (Du et al., 2017). *Botrytis cinerea* infection assay of tomato presented significantly larger necrotic lesions in *MYC2*-RNAi plants than in the wild type (Du et al., 2017). Interestingly, *AtMYC2* negatively regulated resistance to *B. cinerea* and *Fusarium oxysporum* (Song et al., 2014). However, no systematic characterization of bHLH TFs in *D. catenatum* and their functions under biotic and abiotic stresses have been reported yet.

In the present study, 108 *DcbHLH* genes were identified. Phylogenetic analysis of bHLH proteins with Arabidopsis, *O. sativa*, *Phalaenopsis equestris*, and *Apostasia shenzhenica* showed that DcbHLHs were divided into 23 subgroups. Meanwhile, the gene structure, physical and chemical properties, conserved motifs of proteins, and the promoter cis-acting element were analyzed. Besides, the role of JA in enhancing *D. catenatum* resistance to P1 was investigated. At the transcriptome level, the expression of *DcbHLH026*(*MYC2a*)*/027*(*MYC2b*) was significantly changed after MeJA and P1 treatment, indicating that it might be a negative or positive regulator of the JA-mediated plant response to P1. Overexpression of *DcMYC2b* enhanced the tolerance to P1 in transgenic Arabidopsis plants, suggesting that *DcMYC2b*-mediated JA signaling pathway plays a positive regulatory role in *D. catenatum* response to P1. In brief, our study revealed a potential regulatory mechanism of *DcMYC2b* involved in the *D. catenatum* defense against P1, which provides an important theoretical basis for future resistance breeding of *D. catenatum*.

## Materials and methods

### Plant materials and growth conditions

*Dendrobium catenatum* clonal cultivar was placed in a modified 1/2 Murashige and Skoog (MS) medium, cultivated in a growth room with a 12-h light/12-h dark photoperiod, 60% relative humidity, and 25°C temperature.

The Columbia accession (Col-0) of Arabidopsis was used as wild type (WT). Arabidopsis mutants *coi1-1* (Xu et al., 2002), *myc3* (GK445B11; Fernandez-Calvo et al., 2011), and *myc4* (GK491E10; Fernandez-Calvo et al., 2011) were provided by Professor Xie Daoxin of Tsinghua University and Professor Song Susheng of Capital Normal University. *myc2* mutant (SALK_017005c) was obtained from Arashare.^1^ Arabidopsis plants were grown on soil under long-day photoperiod conditions (16-h light/8-h dark).

### Identification and analysis of *bHLH* genes in *Dendrobium catenatum*, *Phalaenopsis equestris*, and *Apostasia shenzhenica*

*Dendrobium catenatum*, *P. equestris*, and *A. shenzhenica* genome sequences were retrieved from NCBI Genome.^2^ The hidden Markov model (HMM) file of the bHLH domain (PF00010) was downloaded from the Pfam database.^3^ The HMMER program was used to search for bHLH proteins with a cut-off *E*-value of 0.001 using PF00010 as a query. We obtained 141 suspected *bHLH* genes in *D. catenatum*, of which the 60 with the lowest e-value were selected to construct species-specific HMM, which was used to check again. The obtained potential *bHLH* genes were further confirmed using Pfam^4^ and SMART databases.^5^ The acquired sequences were submitted to ExPASy^6^ to calculate the molecular weight (MW) and theoretical isoelectric point (pI).

### Bioinformatic analysis of *bHLH* genes

Multiple sequence alignment of bHLH proteins was performed using ClustalW with default parameters. MEGA 7.0 software was used to construct neighbor-joining (NJ) distance trees with the bHLH proteins of *D. catenatum*, *P. equestris*, *A. shenzhenica*, Arabidopsis (Toledo-Ortiz et al., 2003), and *O. sativa* (Li et al., 2006). Evolutionary trees were constructed with the following parameters: 1,000 bootstrap replications, p-distance model or method, and pairwise deletion for gaps or missing data. The phylogenetic tree was subsequently visualized with EvolView.^7^ Subfamily grouping of the DcbHLH proteins was performed according to the classification scheme of the AtbHLH proteins (Heim et al., 2003).

We used GSDS^8^ to predict the number of untranslated regions and coding domain sequences (CDSs) of the *DcbHLH* genes. The conserved motifs were investigated by the MEME version 5.3 online tool.^9^

To investigate putative genes in the promoter regions of *DcbHLH* genes, 1,500-bp genomic DNA sequences upstream of the transcription start site were retrieved and screened against the Plant CARE database.^10^ The gene structure, motif, and composition of cis-acting elements were visualized using the TBtools (Chen et al., 2020a).

### The expression patterns of *DcbHLH* genes

In order to analyze the expression patterns of D*cbHLH* genes, we downloaded the RNA-Seq from the NCBI Sequence Read Archive (SRA; https://www.ncbi.nlm.nih.gov/sra), which contains the data for different organs/tissues (Zhang et al., 2017), drought (Zou et al., 2018), salt (Zhang et al., 2021), and cold stresses (Wu et al., 2016), and *S. delphinii* (MN061040.1, named P1) inoculation assays (Li et al., 2021).

The expression data of different organs/tissues were provided by Zhang et al. (2017), including the expression levels in 10 organs/tissues [leaf (SRR4431601), stem (SRR4431600), root (SRR5722140), green root tip (SRR4431599), white part of the root (SRR4431598), flower bud (SRR4431603), sepal (SRR4431597), labellum (SRR4431602), pollinia (SRR5722145), and gynostemium (SRR4431596)].

For the drought and rewatering experiments, the protocol followed was as follows: watering on the 1st day, simulating drought on the 2nd–7th day, rewatering on the 8th day, and then watering every 2 days at 15:30 h (Zou et al., 2018). The expression data were obtained from the leaves that were collected at both 06:30 and 18:30 h on the 2nd [DR5 (SRR7223299) and DR8 (SRR7223300)], 7th [DR6 (SRR7223298) and DR10 (SRR7223296)], and 9th [DR7 (SRR7223301) and DR15 (SRR7223297)] days, respectively, and at 18:30 h on the 8th day [DR11 (SRR7223295)].

The salt expression data were provided by Zhang et al. (2021). RNA-Seq data included *D. catenatum* plants treated with 250 mM NaCl for 0 h (SRR13986990, SRR13986991, and SRR13986992), 4 h (SRR13987000, SRR13987001, and SRR1387002), and 12 h (SRR13986997, SRR13986998, and SRR1398699) for leaves, and 0 h (SRR13986996, SRR13987005, and SRR13987006), 4 h (SRR13986993, SRR13986994, and SRR13986995), and 12 h (SRR13986989, SRR13987003, and SRR13987004) for roots.

The expression data of leaves under cold stress treatments in 4-month-old *D. catenatum* plants, containing 20°C control (SRR3210630, SRR3210635, and SRR3210636) and 0°C cold acclimations (SRR3210613, SRR3210621, and SRR3210626) for 20 h, were obtained from NCBI provided by Wu et al. (2016).

The P1 inoculation and methyl jasmonate (MeJA) pre-treatment transcriptome data were from our previous study (Li et al., 2021), which consists of four treatments, including CK (no treatment: SRR14635793, SRR14635796, and SRR14635797), JA (pre-treated by MeJA for 4 h: SRR14635790, SRR14635791, and SRR14635792), P1 (inoculated by P1 for 24 h: SRR14635787, SRR14635788, and SRR14635789), and P1 + JA (pre-treated by MeJA for 4 h and then infected by P1 for 24 h: SRR14635786, SRR14635794, and SRR14635795). The expression abundance of *DcbHLH* genes was calculated using the fragments per kilobase of transcript per million fragments mapped (FPKM) values. Using TBtools, we generated a heat map of *DcbHLH* genes (Chen et al., 2020a).

### Pathogen inoculation assays

*Sclerotium delphinii* was grown on the PDA medium at 25°C for 6 days. Five 5-mm agar disks containing mycelia were collected and cultured in 200 ml PDB medium for 6 days at 25°C with constant shaking at 180 rpm. After diluting to 1/2 of the original concentration, it was used for the subsequent inoculation assay of *D. catenatum* and Arabidopsis.

The 4-month-old *D. catenatum* plantlets with the same growth state and size were selected and divided into six groups [CK, P1, Phenidone (Phe), JA, Phe + P1, and JA + P1]. Phenidone decreased endogenous JA synthesis by inhibiting the activity of 13-lipoxygenase (LOX) in plants (Bruinsma et al., 2010). The plantlets were pre-treated with sterile water (mock) or a solution containing Phe (16 mmol/L) or MeJA (100 μ*mol*/L) for 4 h. After that, they were washed with sterile water and put into a sterile bottle with sterile water. Among them, P1, Phe + P1, and JA + P1 were evenly sprayed with 2 ml of mycelia suspensions (the others were spotted with 0.25% ethanol solution). The infection ratings and disease index were calculated as described previously at each time point (0, 24, 30, 38, 48, and 72 h; Li et al., 2021). The infection experiment was repeated three times with 10 plantlets each (30 plantlets per infection). The leaves of each treatment were collected at 24 h post-inoculation (hpi) for subsequent analysis.

The leaves with relatively consistent growth status and size in Arabidopsis Col-0, mutants *coi1-1*, *myc2*, *myc3*, and *myc4*, and transgenic lines grown for 20 days were placed on tetrad Petri dishes containing filter paper moistened with sterile water, and each leaf was inoculated with 5 μl of sterile water (mock) or mycelia suspensions in the middle position on the front of each leaf, with three replicates for at least 10 leaves in each experiment. The size of the leaf lesion was calculated at 48 hpi using ImageJ.^11^

### Determination of JA content using LC–MS/MS

The content of JA, (3R,7S)-jasmonoyl-L-isoleucine (JA-Ile), and 12-oxophytodienoic acid (OPDA) were measured by LC–MS/MS. In short, the leaves of *D. catenatum* preserved at ultralow temperature after inoculating by P1 for 24 h were ground with mixer mil (30 Hz, 1 min). Then, 50 mg of leaf samples were extracted with methanol/water/formic acid (15:4:1, V/V/V) at 4°C (vortex, 10 min), and then centrifuged at 12,000 rpm for 5 min. The supernatant was evaporated and concentrated with nitrogen at room temperature, reconstituted in 100 μl of 80% (*v/v*) methanol, and filtered (SCAA-104, 0.22 μm pore size; ANPEL, Shanghai, China). It was placed in the injection bottle for LC–MS/MS (Ultra-performance liquid chromatography (UPLC), ExionLC™ AD, https://sciex.com.cn/; and Tandem mass spectrometry (MS/MS), QTRAP® 6,500+, https://sciex.com.cn/). Three replicates of each assay were performed. Based on the MWDB (Metware database) constructed by the standard, the mass spectrometry data were analyzed qualitatively. Multiple reaction monitoring (MRM) of triple quadrupole mass spectrometry and the internal standard method were used for quantitative analysis. Analyst 1.6.3 software was used to process MS data.

### RNA extraction and quantitative real-time PCR

The total RNA was extracted using the MiniBEST Plant RNA Extraction Kit (TaKaRa, Japan). The cDNA was reverse-transcribed with the PrimerScript RT Enzyme Mix I kit (TaKaRa, Japan). qPCR analysis was performed with SYBR® Premix Ex Taq II (TaKaRa, Japan) on CFX96 Touch™ Real-Time PCR System (BIO-RAD, United States). Three independent biological replicates were performed on each analysis with three technical replicates. The *DcACTIN* was used as the internal control gene according to the previous study (Li et al., 2021). The relative expression levels were evaluated using the 2-ΔΔCT method [3]. The gene-specific primers were designed by Primer Premier 5 (Supplementary Table S1).

### Protein secondary structure and tertiary structure prediction

Online software SOPMA 2.0^12^ was used to predict the secondary structure of the four DcbHLH IIIe proteins, that is, DcbHLH025/026/027/028 proteins. SWISS-MODEL online homology modeling^13^ was applied to predict the tertiary structure of each of the above four proteins, and the predicted protein tertiary structure models were assessed for Model Credibility using SAVES online software.^14^ DcbHLH025/026/027/028 was renamed as DcMYC2d/2a/2b/2c, respectively, which is used throughout the rest of the article.

### Subcellular localization of *DcMYC2a* and DcMYC2b

The CDS of *DcMYC2a* and *DcMYC2b* were cloned into the plant expression vector pMDC43 to construct *35S: DcMYC2a:GFP* and *35S: DcMYC2b:GFP* fusion construct, respectively. Subsequently, the control plasmid (empty vector) and fusion plasmids were transiently expressed in tobacco leaves. After 3 days of post-infiltration, the GFP fluorescence was observed using a confocal microscope (Zeiss, LSM 880) with 488 and 594 nm argon lasers.

### Generation of transgenic plants and transgene analysis

Arabidopsis (Col-0) plants were used for *Agrobacterium tumefaciens*-mediated transformation to generate *DcMYC2a/2b*-overexpressing (*DcMYC2a/2b*-OE) plants. The recombinant plasmids *pMDC43-DcMYC2a-GFP* and *pMDC43-DcMYC2b-GFP* were transformed into *A. tumefaciens* GV3101, which was used to transform Arabidopsis plants by the floral-dip method. Regenerated seedlings were selected on MS medium with 20 mg.L^−1^ hygromycin. The expression of *DcMYC2a/2b* in transgenic Arabidopsis plants was analyzed by quantitative real-time PCR (qRT-PCR). Homozygous T3 generation transgenic lines were used for further experiments.

## Results

### Identification and characterization of *DcbHLH*, *PebHLH*, and *AsbHLH* genes

We conducted a genome-wide search for *D. catenatum*, *P. equestris*, and *A. shenzhenica*, which together belong to the Orchidaceae family. A total of 108 DcbHLH, 83 AsbHLH, and 109 PebHLH proteins were obtained after removing redundant proteins. To further characterize these bHLH proteins, we analyzed the physicochemical properties of these proteins (Table 1; Supplementary Tables S2, S3). The amino acids of DcbHLH ranged from 85 (DcbHLH089) to 662 (DcbHLH031) in length and from 9534.7 (DcbHLH089) to 74539.8 (DcbHLH031) in molecular weight, the amino acid length of PebHLH ranged from 85 (PebHLH086) to 916 (PebHLH091) and the molecular weight size from 9578.7 (PebHLH086) to 100520.6 (PebHLH091), and the amino acid length of AsbHLH ranged from 67 (AsbHLH042) to 687 (AsbHLH026) and the molecular weight size from 7702.9 (AsbHLH042) to 74504.6 (AsbHLH026). The pI ranged from 4.21 (DcbHLH015) to 11.93 (DcbHLH090) for the DcbHLH proteins, from 4.47 (AsbHLH009) to 12.28 (AsbHLH042) for the AsbHLH proteins, and from 4.15 (PebHLH011) to 12.13 (PebHLH087) for the PebHLHs. The large differences in molecular weight, amino acid length, and pI among the DcbHLH, PebHLH, and AsbHLH members indicated that the *DcbHLH*, *PebHLH,* and *AsbHLH* gene family members may have undergone a long historical evolution and participated in different biological processes.

**Table 1:** Characteristics of *bHLH* genes in *Dendrobium catenatum*.

No.	Gene ID	Rename	Clade	Protein (aa)	pI	MW (Da)
1	LOC110112576	DcbHLH001	Ia	193	8.76	21446.7
2	LOC110108487	DcbHLH002	Ia	235	9.78	25982.7
3	LOC110111619	DcbHLH003	Ia	268	7.24	29804.5
4	LOC110098508	DcbHLH004	Ia	381	4.62	41659.4
5	LOC110107102	DcbHLH005	Ia	312	6.52	35170.6
6	LOC110116721	DcbHLH006	Ia	291	5.94	33087.6
7	LOC110115756	DcbHLH007	Ia	324	9.00	36507.5
8	LOC110096422	DcbHLH008	Ia	283	6.88	32378.8
9	LOC110116474	DcbHLH009	Ia	319	6.44	35359.8
10	LOC110112336	DcbHLH010	Ib	275	8.58	30589.8
11	LOC110099884	DcbHLH011	Ib	290	4.27	31468.4
12	LOC110101583	DcbHLH012	Ib	290	4.24	31763.4
13	LOC110105593	DcbHLH013	Ib	180	9.42	19967.0
14	LOC110112688	DcbHLH014	II	338	5.71	38160.1
15	LOC110114520	DcbHLH015	II	245	4.21	26967.0
16	LOC114580710	DcbHLH016	II	364	4.96	40612.8
17	LOC110112097	DcbHLH017	IIIb	531	4.75	56541.0
18	LOC110096203	DcbHLH018	IIIb	292	4.49	32404.7
19	LOC110109085	DcbHLH019	IIIc	252	4.67	28218.7
20	LOC110103241	DcbHLH020	IIId	558	7.43	61580.4
21	LOC110094094	DcbHLH021	IIId	499	6.11	55244.4
22	LOC110116682	DcbHLH022	IIId	422	6.38	45803.3
23	LOC110099101	DcbHLH023	IIId	419	6.28	45777.2
24	LOC110113808	DcbHLH024	IIId	381	7.36	42301.5
25	LOC110094435	DcbHLH025(MYC2d)	IIIe	462	4.96	51217.4
26	LOC110116479	DcbHLH026(MYC2a)	IIIe	651	5.01	72046.3
27	LOC110114462	DcbHLH027(MYC2b)	IIIe	636	5.90	69408.2
28	LOC110092865	DcbHLH028(MYC2c)	IIIe	656	6.27	72002.0
29	LOC110111891	DcbHLH029	IIIf	644	5.30	73817.4
30	LOC110114654	DcbHLH030	IIIf	653	4.91	74171.6
31	LOC110097687	DcbHLH031	IIIf	662	5.55	74539.8
32	LOC110093619	DcbHLH032	IVa	328	7.57	35773.4
33	LOC110110298	DcbHLH033	IVa	364	7.50	39452.4
34	LOC110115484	DcbHLH034	IVa	333	5.58	37072.8
35	LOC110096269	DcbHLH035	IVb	223	6.63	24441.3
36	LOC110095837	DcbHLH036	IVb	353	6.85	38503.4
37	LOC110098909	DcbHLH037	IVc	213	5.28	24476.7
38	LOC110102219	DcbHLH038	IVc	233	7.37	25626.2
39	LOC110107046	DcbHLH039	IVc	243	6.93	27259.8
40	LOC110093741	DcbHLH040	IVc	152	9.97	17380.0
41	LOC110116147	DcbHLH041	IVd	439	5.33	49294.3
42	LOC110094813	DcbHLH042	IVd	510	7.43	57490.0
43	LOC110094726	DcbHLH043	IVd	243	4.77	27329.5
44	LOC110107826	DcbHLH044	IVd	254	10.06	27399.7
45	LOC110107963	DcbHLH045	Va	471	6.66	51719.6
46	LOC110114469	DcbHLH046	Va	348	6.38	38657.0
47	LOC110106259	DcbHLH047	Va	285	5.97	32037.3
48	LOC110107930	DcbHLH048	Vb	256	10.38	28079.4
49	LOC110094861	DcbHLH049	Vb	249	7.85	26969.3
50	LOC110115493	DcbHLH050	Vb	196	9.08	20728.3
51	LOC110112072	DcbHLH051	Vb	325	4.83	35270.2
52	LOC110113204	DcbHLH052	VIIa	658	6.75	71440.4
53	LOC110113329	DcbHLH053	VIIa	311	6.61	34212.1
54	LOC110108343	DcbHLH054	VIIa	475	8.67	53109.6
55	LOC110099714	DcbHLH055	VIIa	490	7.27	54630.9
56	LOC110113833	DcbHLH056	VIIa	490	6.97	54560.7
57	LOC110095032	DcbHLH057	VIIb	394	7.14	42672.7
58	LOC110109507	DcbHLH058	VIIa	305	9.08	34112.9
59	LOC110100961	DcbHLH059	VIIa	172	5.33	19135.9
60	LOC110105526	DcbHLH060	VIIa	133	7.91	14862.0
61	LOC114578594	DcbHLH061	VIIa	178	4.48	19728.3
62	LOC110098199	DcbHLH062	VIIb	213	8.47	23152.0
63	LOC110094287	DcbHLH063	VIIb	195	10.02	21677.8
64	LOC110098270	DcbHLH064	VIIb	157	10.78	17334.1
65	LOC110112441	DcbHLH065	VIIb	304	6.51	34517.8
66	LOC110113891	DcbHLH066	VIIb	361	6.32	40668.3
67	LOC110101026	DcbHLH067	VIIb	180	9.70	19841.3
68	LOC110111081	DcbHLH068	VIIc	356	5.04	39904.1
69	LOC110104845	DcbHLH069	VIIc	275	4.41	30879.1
70	LOC110095998	DcbHLH070	VIIc	310	4.45	34760.2
71	LOC110110399	DcbHLH071	VIIc	294	6.35	33416.4
72	LOC110112285	DcbHLH072	IX	347	6.10	37393.1
73	LOC110108563	DcbHLH073	IX	377	6.72	39787.5
74	LOC110114625	DcbHLH074	IX	398	8.13	43448.0
75	LOC110106166	DcbHLH075	IX	361	7.79	40249.6
76	LOC110103817	DcbHLH076	X	366	6.17	39724.9
77	LOC110107318	DcbHLH077	X	340	7.32	37919.2
78	LOC110107832	DcbHLH078	X	134	8.71	15186.2
79	LOC110114754	DcbHLH079	X	343	6.16	38425.2
80	LOC110098511	DcbHLH080	X	308	4.62	34456.1
81	LOC110107031	DcbHLH081	X	355	6.29	39623.6
82	LOC110115095	DcbHLH082	X	225	4.96	23831.3
83	LOC110093188	DcbHLH083	X	298	7.14	33028.6
84	LOC110107294	DcbHLH084	X	334	7.24	36713.7
85	LOC110116300	DcbHLH085	X	368	5.72	41260.0
86	LOC110107413	DcbHLH086	X	275	6.78	30447.3
87	LOC110110081	DcbHLH087	X	163	8.66	17755.0
88	LOC110108630	DcbHLH088	X	89	9.36	10113.2
89	LOC110110529	DcbHLH089	X	85	8.52	9534.7
90	LOC110097090	DcbHLH090	X	165	11.93	18075.2
91	LOC110093428	DcbHLH091	XI	459	6.42	47032.9
92	LOC110111433	DcbHLH092	XI	412	4.96	43403.1
93	LOC110095526	DcbHLH093	XI	320	6.17	34348.2
94	LOC110101829	DcbHLH094	XI	496	5.56	53461.1
95	LOC110099461	DcbHLH095	XII	382	6.26	41065.1
96	LOC110114258	DcbHLH096	XII	325	5.27	35929.4
97	LOC110109277	DcbHLH097	XII	420	8.46	46740.3
98	LOC110114447	DcbHLH098	XII	482	6.53	53020.0
99	LOC110096494	DcbHLH099	XII	321	6.26	36376.9
100	LOC110095482	DcbHLH100	XII	272	5.45	29292.4
101	LOC110114740	DcbHLH101	XII	261	6.52	28770.7
102	LOC110099214	DcbHLH102	XII	211	5.02	23847.6
103	LOC110104725	DcbHLH103	XII	383	6.12	41864.7
104	LOC110102342	DcbHLH104	XII	533	8.45	58799.1
105	LOC110107722	DcbHLH105	XII	362	8.90	39857.7
106	LOC110100649	DcbHLH106	XII	453	6.51	49016.8
107	LOC110107433	DcbHLH107	XII	284	6.24	32239.7
108	LOC110111863	DcbHLH108	XII	263	5.74	29734.5

### Phylogenetic analysis of *bHLH* genes

**Figure 1:**
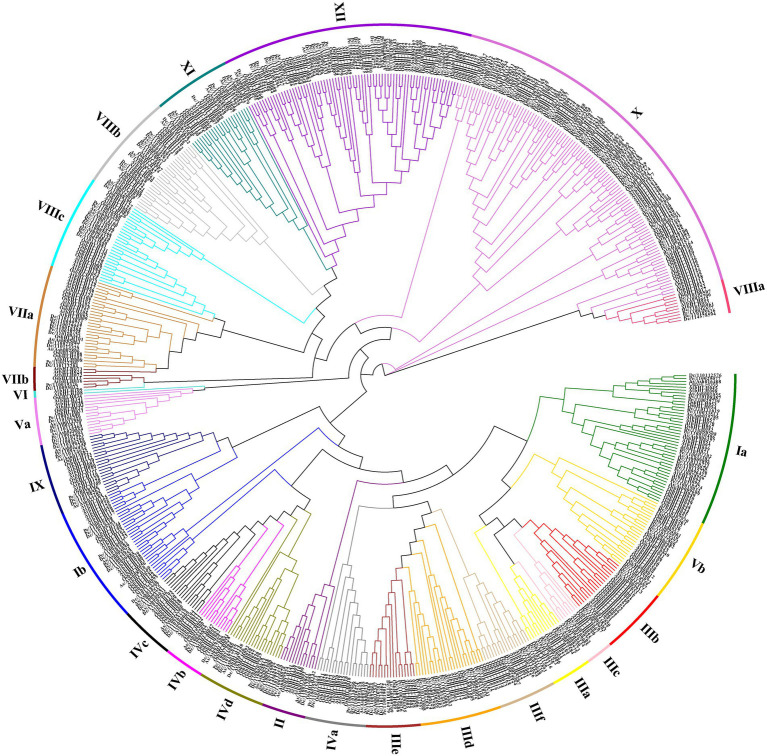
Phylogenetic analysis of the *basic helix–loop–helices* (bHLHs) from *Dendrobium catenatum*, *Phalaenopsis equestris*, *Apostasia shenzhenica*, *Arabidopsis*, and *Oryza sativa* genome. The neighbor-joining (NJ) tree was drawn using MEGA 7.0 with 1,000 bootstrap replicates. Dc, *D. catenatum*; Pe, *P. equestris*; As, *A. shenzhenica*; At, *Arabidopsis*; and Os, *O. sativa.*

To better study the classification and evolutionary relationships of the DcbHLH proteins and to further predict the function of the DcbHLH proteins, we constructed a phylogenetic tree based on bHLH protein sequences from five species (Arabidopsis, *O. sativa*, *D. catenatum*, *P. equestris*, and *A. shenzhenica*) using the neighbor-joining (NJ) method (Figure 1). According to the clade support values and the classification of Arabidopsis, the 108 DcbHLH proteins were clustered into 23 subgroups, and the other two species belonging to the Orchidaceae cluster similarly to *D. catenatum*, where 109 AsbHLH proteins cluster into 24 subgroups and 83 PebHLH proteins cluster into 24 subgroups. The results showed that the largest number of members of the X subgroup contained 12 DcbHLH proteins, 12 AsbHLH proteins, and 21 PebHLH proteins. Interestingly, none of the DcbHLH, AsbHLH, or PebHLH proteins clustered in subgroup VI, where DcbHLH was also not identified in subgroup IIIa. It is possible that *D. catenatum* has lost the genes of IIIa and VI subgroups during evolution. Arabidopsis *bHLH* genes, belonging to four subgroups of the III subfamily, are involved in JA signal transduction pathways, including subgroup IIIe which positively regulates JA response, subgroup IIId which negatively regulates JA response, subgroup IIIf which is involved in JA-mediated anthocyanin synthesis and epidermal hair initiation, and subgroup IIIb which is associated with JA-induced freezing tolerance (Heim et al., 2003). We predicted that the *DcbHLH*, *PebHLH*, and *AsbHLH* genes, which are in these four subgroups, might also be involved in the JA signal transduction pathway.

### Conserved motif, gene structure, and Cis-acting element analysis of *DcbHLH* genes

**Figure 2:**
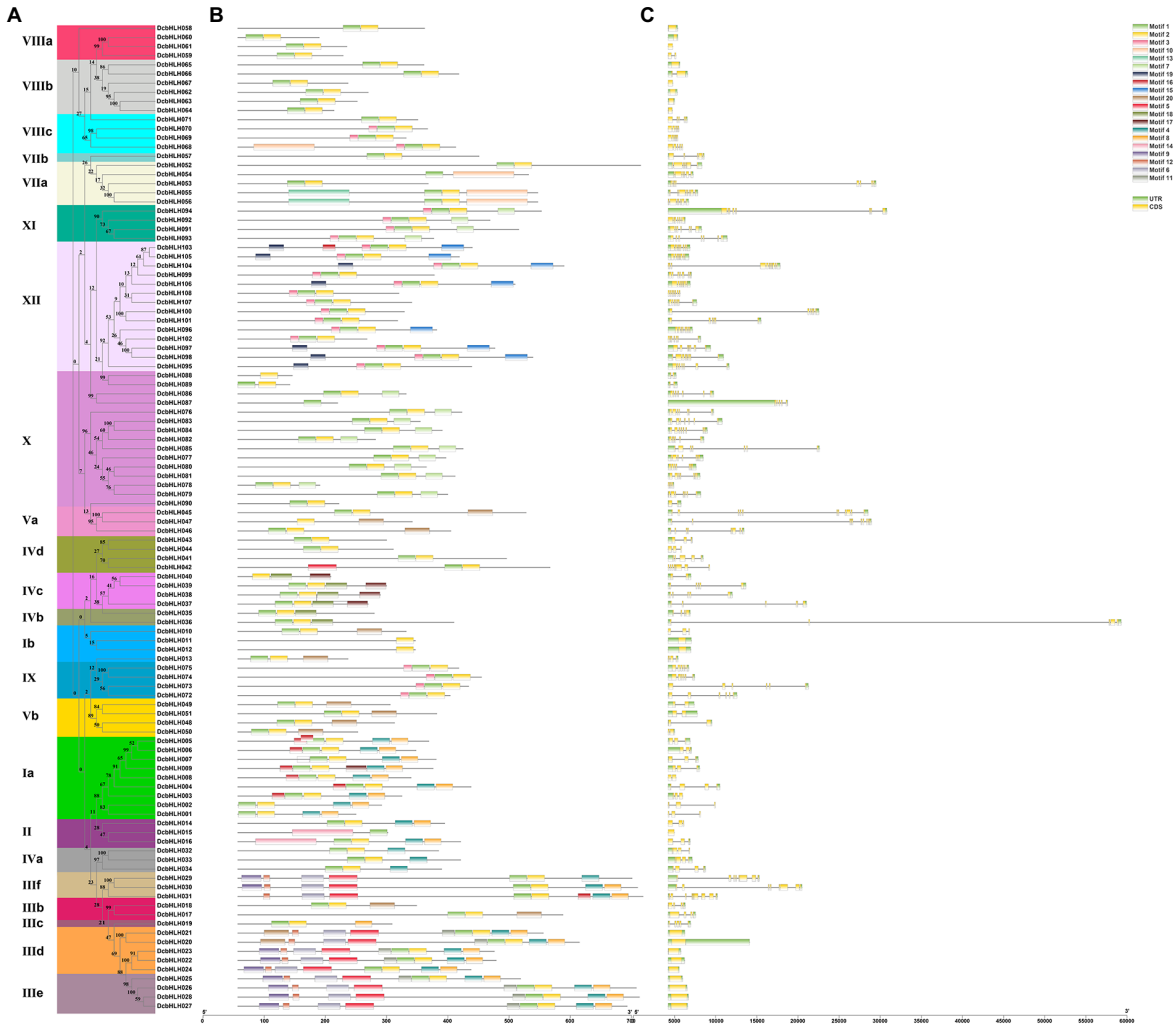
Phylogenetic relationships, architecture of conserved protein motifs, and gene structure in *bHLHs* of *Dendrobium catenatum*. **(A)** The phylogenetic tree of bHLH proteins. It was constructed according to the NJ method by MEGA 7.0 with 1,000 bootstrap replicates. Different color boxes represent different clades. **(B)** The conserved motif composition of bHLH proteins. Solid boxes of different colors represent different motifs, and the legend is on the right side of the figure. **(C)** UTR-coding domain sequence (CDS) structure of *bHLH* genes. The green box represents UTRs, and the yellow box represents CDSs.

In order to verify the accuracy of phylogenetic analysis and further study the structure of DcbHLH proteins, we identified 20 conserved motifs using the MEME program (Figures 2A,B). DcbHLH proteins in the same subgroup have similar conserved motifs, which indicate that these DcbHLH proteins may have similar functions. The vast majority of DcbHLH proteins contain motif 1 and motif 2, suggesting that the *DcbHLH* genes are functionally similar in some ways. In addition, some subfamilies contain specific motifs. For instance, motif 6, motif 9, motif 11, and motif 12 are unique to subfamily III. It is possible that these motifs are involved in JA signal transduction pathways in the plant.

Exon-intron structural diversity is considered to play an important role in the evolution of *bHLH* genes. We mapped the gene structure based on the sequence information of the *DcbHLH* gene and its CDS (Figures 2A,C). The number of introns contained in the *DcbHLH* gene ranges from 0 to 10, of which 20 *DcbHLH* genes do not contain introns, nine *DcbHLH* genes contain one intron, and the other genes contain two or more introns. Members belonging to the same subgroups had similar patterns of intron-exon distribution. For instance, members belonging to subgroups IIId and IIIe contained no intron, but all contained one exon, and members belonging to subgroup IIIf contained both seven introns and eight exons.

Many *bHLH* genes play important roles in plant growth and development as well as in the response to various stresses (Mao et al., 2017; Hao et al., 2021). In order to further predict the function of the *DcbHLH* genes, we made a prediction of cis-acting elements for the sequence 1,500 bp upstream of the start codon of the *DcbHLH* genes (Supplementary Figure S1), and cis-acting elements can be classified into three categories according to functions. The first category comprises phytohormone-responsive elements, including methyl jasmonate (MeJA-responsive, 13.79%), abscisic acid (ABA-responsive, 8.69%), salicylic acid (SA-responsive, 1.75%), gibberellins (GA-responsive, 2.93%), and auxins-responsive (2.57%) elements, and this category consists of TGACG-motif, CGTCA-motif, ABRE, TCA-element, TATC-box, P-box, GARE-motif, TGA-element, TGA-box, AuxRE, and AuxRR-core. The second category belongs to the growth and development class of elements (8.08%), including cell differentiation, circadian control, cell cycle regulation, meristem expression, and flavonoid synthesis, and this class consists of ARE, AT-rich sequence, HD-Zip-1, RY-element, GCN4_motif, AACA_motif, circadian, MSA-like, CAT-box, and MBSI. The third category comprises stressor elements that are involved in adaptation to adversity and include light-responsive (45.83%), anaerobic induction (6.22%), low-temperature responsive (2.47%), drought-inducible (2.11%), defense and stress-responsive (2.06%), anoxic specific induction (0.41%), and wound-responsive (3.09%) elements; this category consists of ACE, G-box, GT1-motif, 3-AF1 binding site, Sp1, AAAC-motif, Box 4, ATC-motif, ATCT-motif, CAG-motif, GATA-motif, Box II, TCT-motif, chs-CMA1a, Pc-CMA2c, LAMP-element, I-box, GA-motif, Gap-box, TCCC-motif, chs-Unit 1 m1, L-box, LS7, AE-box, ARE, LTR, MBS, TC-rich repeats, GC-motif, and WUN-motif. Different types and numbers of regulatory elements were identified in the promoter regions of different *DcbHLH* genes, indicating that *DcbHLH* genes might have different functions in stress resistance, growth, and development.

### The expression patterns of *DcbHLH* genes

**Figure 3:**
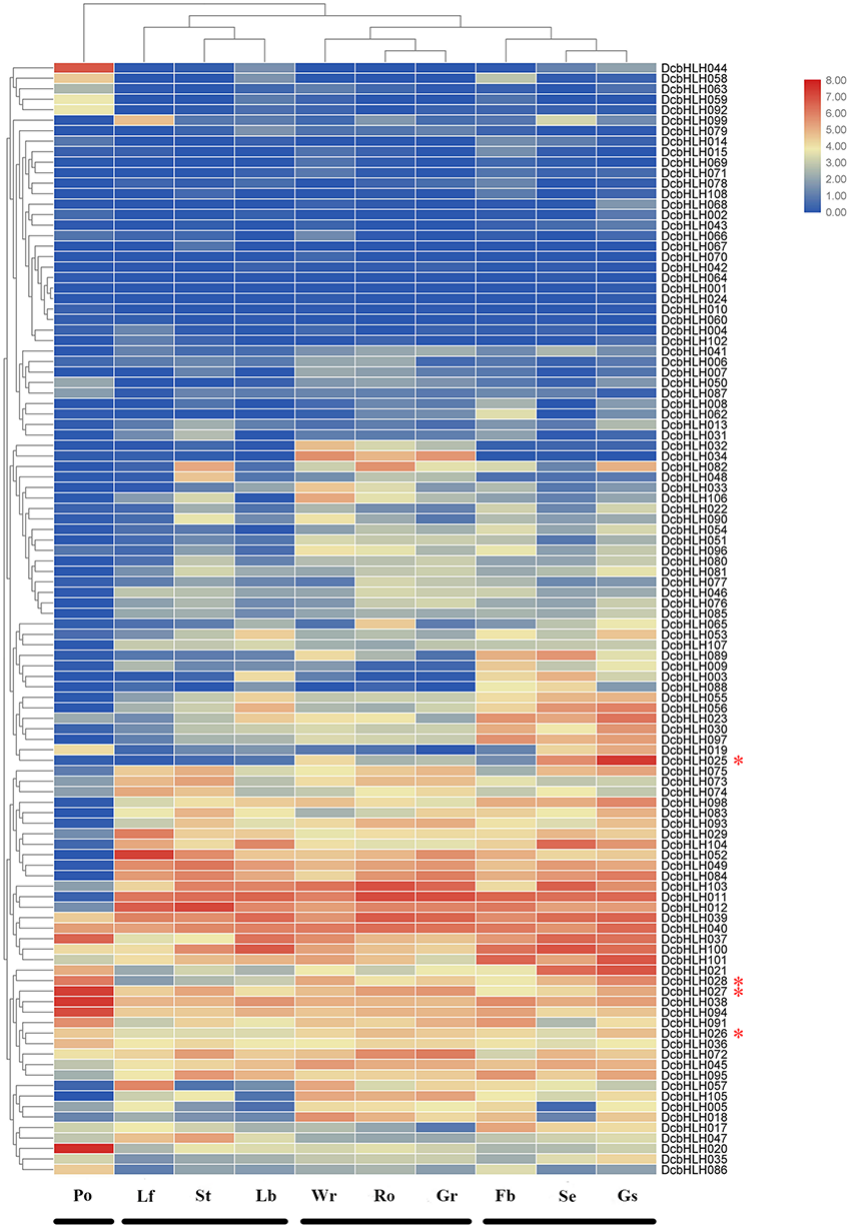
Relative expression analysis of the *DcbHLH* genes in different tissues. Expression patterns of *DcbHLH* genes in 10 *Dendrobium catenatum* tissues and organs. Lf, leaf; Ro, root; Gr, green root tip; Wr, the white part of the root; St, stem; Fb, flower bud; Se, sepal; Lb, labellum (lip); Po, pollinia; and Gs, gynostemium (column).

We analyzed the temporal and spatial expression patterns of 108 *DcbHLH* genes in different tissues or organs (leaf, stem, root, green root tip, white part of the root, flower bud, sepal, labellum, pollinia, and gynostemium; Figure 3; Supplementary Table S4). The expression of some *DcbHLH* genes in various tissues or organs is very low or basically not expressed, such as *DcbHLH001/064/070*, which indicates that these genes may not be necessary for these tissues or organs to perform their functions at this stage. Some genes also show high expression in various tissues or organs, such as *DcbHLH039/040*. Most genes are highly expressed in one or more tissues or organs. In addition, the expression of *DcbHLHs* can be roughly divided into four groups based on the similarity of gene expression in different tissues and organs: I (pollinia), II (leaf, stem, and labellum), III (root, green root tip, and white part of the root), and IV (flower bud, sepal, and gynostemium). The above results showed that the *DcbHLHs* family is widely involved in plant growth and development.

**Figure 4:**
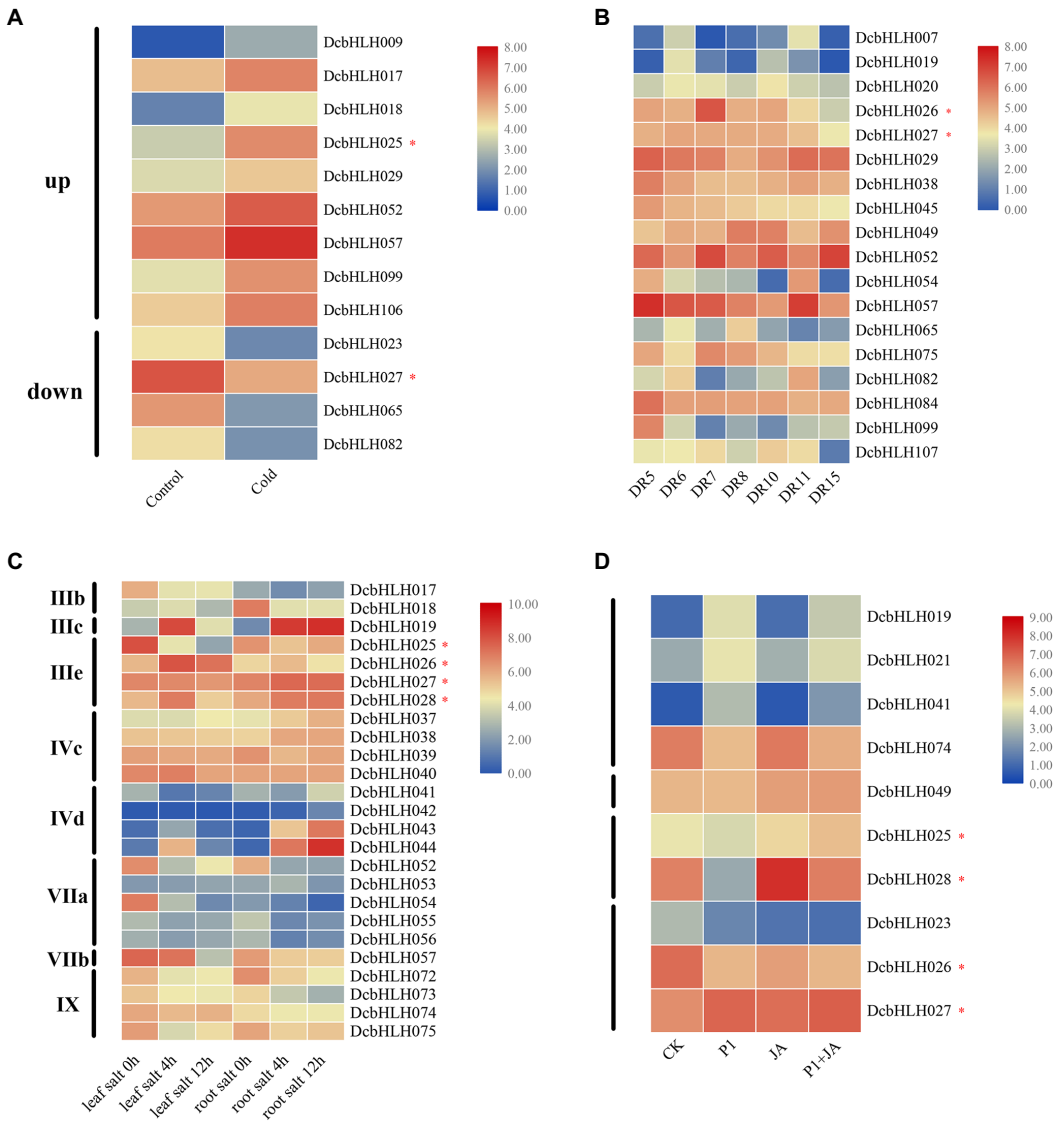
Relative expression analysis of the *DcbHLH* genes under different stress conditions. **(A)** Low-temperature treatment (CK: 20°C, 20 h; Cold: 0°C, 20 h). **(B)** Drought treatment [Drought stage: DR5/DR6 (the next day 06:30 and 18:30 h), Dr7/DR8 (06:30 and 18:30 h on the seventh day)]; Rehydration stage: DR10 (18:30 h on the eighth day), and DR11/DR15 (06:30 and 18:30 h on the ninth day). **(C)** Salt stress treatment. Leaf salt 0/4/12 h: Leaf treated with 250 mM NaCl for 0/4/12 h. Root salt 0/4/12 h: Root treated with 250 mM NaCl for 0/4/12 h. **(D)** Expression profiles of *DcbHLH* genes to *S. delphinii* after pre-treatment with MeJA (CK: control. P1: 24 h after post-inoculation with *S. delphinii*. JA: pre-treated with MeJA for 4 h and then inoculated with sterile distilled water for 24 h. P1 + JA: pre-treated with MeJA for 4 h and then inoculated with *S. delphinii* for 24 h).

In the low-temperature treatment (Figure 4A; Supplementary Table S5), compared with the control group, the expression of *DcbHLH009/017/018/025*(*MYC2d*)*/029/052/057/099/106* was upregulated significantly, while the expression of *DcbHLH023/027*(*MYC2b*)*/065/082* was downregulated significantly. Among them, *DcbHLH* IIId subgroup member *DcbHLH017* was highly homologous with Arabidopsis *Inducer of CBF Expression 1* (*AtICE1*), which is a positive regulator of plant resistance to cold stress (Chinnusamy et al., 2003), indicating that *DcbHLH017* is likely to positively regulate plant cold resistance.

In the drought–rewatering assay, most genes showed non-significant trends, but a small number of genes showed significant changes (Figure 4B; Supplementary Table S6). At the DR5-DR8 stage (continuous drought stage), the expression levels of *DcbHLH019/026* (*MYC2a*) were upregulated first and then decreased, those of *DcbHLH029/038/045/054/057/082/084/099* were downregulated, that of *DcbHLH049* was upregulated gradually, that of *DcbHLH052* downregulated first, rapidly upregulated, and then sharply downregulated, whereas the expression of *DcbHLH065* showed the opposite trend. At the DR10-DR15 stage (the rewatering stage), the expression of *DcbHLH007/020/026*(*MYC2a*)*/029/082/107* was upregulated first and then downregulated, while *DcbHLH049* showed the opposite trend. The expressions of *DcbHLH027*(*MYC2b*)*/065/075* gradually decreased, and the expression of *DcbHLH052* was upregulated first, then decreased, and then increased, while *DcbHLH054/057* showed the opposite trend.

In the high salt environment (Figure 4C; Supplementary Table S7), we found that there were several subgroups whose expression in leaves or roots changed significantly after plants were subjected to salt stress, including subgroups IIIb, IIIc, IIIe, IVc, IVd, VIIa, VIIb, and IX. In particular, the expression of the genes of subgroup IIIe, that is, *DcbHLH025*(*MYC2d*)*/026*(*MYC2a*)*/027*(*MYC2b*)*/028*(*MYC2c*), which are related to JA signaling pathway, was found to be significantly altered. These results suggested that some subgroups of DcbHLH TFs may play important roles in plant resistance to salt stress and that the JA signal transduction pathway may have been induced after plants were subjected to salt stress. The expression of the *DcbHLH* gene in the stem and leaves of *D. catenatum* under salt stress conditions is significantly different (Figures 3, 4C), which may be due to the tissue specificity of gene expression.

Given that some *bHLH* genes play pivotal roles in plant response to biotic stress (Dombrecht et al., 2007; Hao et al., 2021), we designed a set of experimental conditions to determine their functions after infection with P1 and treatment with the resistance hormone JA (Li et al., 2021). Compared with the CK group, the expression of some *DcbHLH* genes in the three treatment samples (P1, JA, and P1 + JA) changed significantly (Figure 4D; Supplementary Table S8). In addition, *DcbHLH019/021/041/074* showed significant changes in the expression following P1 induction but were unaffected by exogenous JA, indicating that these genes were specifically induced by P1. Some genes, such as *DcbHLH049*, were induced only by JA, suggesting that these genes did not respond to P1, which might be involved in specific resistance or development pathways triggered by high concentrations of JA. Some genes, such as *DcbHLH025*(*MYC2d*)*/028*(*MYC2c*), were upregulated by JA but repressed by P1, suggesting that these two genes might be involved in JA-regulated growth and development pathways. The transcript levels of *DcbHLH023/026*(*MYC2a*) were significantly downregulated in P1, JA, and P1 + JA treatment samples, which indicated that P1 might be a negative regulator of the JA-mediated plant response. In contrast to the *DcMYC2a*, the *DcbHLH027* (*MYC2b*) was significantly upregulated in all treatment samples, indicating that it may play a positive regulatory role.

### JA enhanced the resistance of *Dendrobium catenatum* to P1

**Figure 5:**
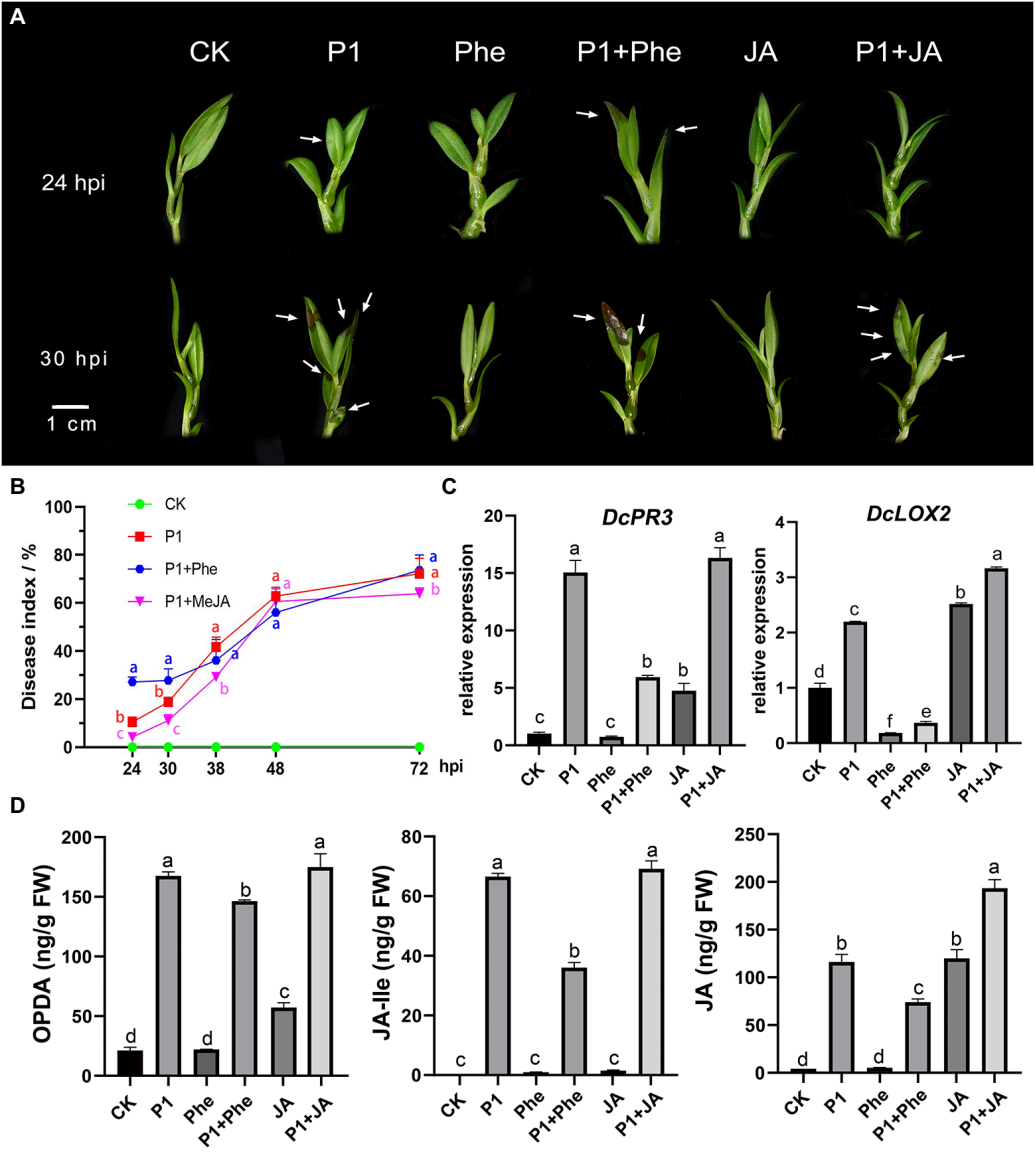
Enhanced resistance of *D. catenatum* to P1 infection by JA. **(A)** Disease symptoms of *D. catenatum* plantlets pre-treated with Phe (16 mmol/L) or MeJA (100 μmol/L) following inoculation with *S. delphinii* at 24 and 30 hpi. Arrows point to the lesion. Bar = 1 cm. **(B)** Disease indexes line charts of the MeJA- or Phe-pre-treated *D. catenatum* were determined from 24 hpi (1 dpi) to 72 hpi (3 dpi). The values are the mean ± SD; *n* = 10. “abc” indicates a significant difference at *p* = 0.05. **(C)** The expression of important downstream gene *DcPR3*, *DcLOX2* of JA signaling pathway under different treatments through RT-qPCR assay. CK: control. P1: 24 h post-inoculation with *S. delphinii.* JA: pre-treated with MeJA for 4 h and then inoculated with sterile distilled water for 24 h. P1 + JA: pre-treated with MeJA for 4 h and then inoculated with *S. delphinii* for 24 h. Phe: pre-treated with MeJA for 4 h and then inoculated with sterile distilled water for 24 h. Phe + P1: pre-treated with Phe for 4 h and then inoculated with *S. delphinii* for 24 h. The actin gene of *D. catenatum* was used as an internal control. The error bars indicate SD from three independent experiments. The “abcdef” showed a significant difference at *p* = 0.05 compared with the CK. **(D)** Content of oxophytodienoic acid (OPDA), JA-lle, and JA in the leaves of *D. catenatum* after inoculation with *S. delphinii* for 24 h. The abscissa represents the different treatments, and the ordinate represents the content of the multitest substance (ng/g FW). “abcdef” indicates a significant difference at *p* = 0.05.

In order to explore the effect of JA on the resistance of *D. catenatum* to P1 infection, we carried out a P1 inoculation experiment. Different responses to the disease infection were observed in MeJA- and phe-pre-treated, and control plantlets (Figures 5A,B; Supplementary Table S9). At 24 hpi, the symptoms, including water-stained and brown necrotic lesions, were much more severe in the plantlets pre-treated with phe than in the control plantlets, whereas the plants pre-treated with exogenous MeJA remained disease-free (Figures 5A,B; Supplementary Table S9). The disease index increased with prolonged hpi, and the MeJA-pre-treated plantlets showed the slowest disease development in early stage (24–30 hpi), showing a certain resistance to P1, while the plantlets pre-treated with phe showed a rapid development. The plantlets treated with the above three treatments (P1, Phe + P1, and JA + P1) were in the same disease situation at about 38 hpi. To better understand the mechanism of *D. catenatum* resistance to P1, we measured the endogenous JA content in *D. catenatum*, including 12-oxophytodienoic acid (OPDA), the receptor-active ligand (3R,7S)-jasmonoyl-L-isoleucine (JA-Ile), and JA at 24 hpi (Figure 5D; Supplementary Table S10). The results showed that a large amount of endogenous JAs was synthesized after P1 infection, which suggested that P1 activated the plant JA signaling pathway. The content of all three JAs was significantly decreased in the treatment P1 + Phe, but not completely suppressed, indicating that phenidone only partially suppresses the activity of LOX and plants still show partial JA-related resistance. There was no significant difference in OPDA content between P1 and P1 + JA samples, indicating that they had approximately equal rates of endogenous JA synthesis, and the difference in JA content between these two samples might be caused by exogenous MeJA demethylation. Interestingly, the levels of JA-lle, the active form of JA in planta, were not significantly different between P1 and P1 + JA samples. It is possibly due to the fact that the catalytic activity of Jasmonoyl-isoleucine synthetase (JAR1), which catalyzes JA synthesis of JA-lle, reached its maximum at an earlier time point, leading to the stronger resistance of JA + P1 sample at the pre-stage and preventing pathogen infiltration. To further explore the reasons for the differences in various treatments, we conducted qRT-PCR of the marker genes of the JA signaling pathway. The results indicated that the expression of *DcPR3* was significantly induced by P1 infection or exogenous JA treatment (Figure 5C), while that of *DcPR3* was inhibited in the samples pre-treated by Phe. The expression of *DcLOX2* was significantly different in P1, JA, and P1 + JA treatments compared to CK, and it was significantly inhibited in Phe and Phe + P1 samples but significantly induced in P1, JA, and P1 + JA samples. More importantly, the expression level of *DcLOX2* was significantly higher in the P1 + JA sample than in the P1 sample. These results suggest that the JA pathway plays a key role in the resistance of *D. catenatum* to P1.

### Protein structure prediction and subcellular localization of *DcbHLH* IIIe genes

**Figure 6:**
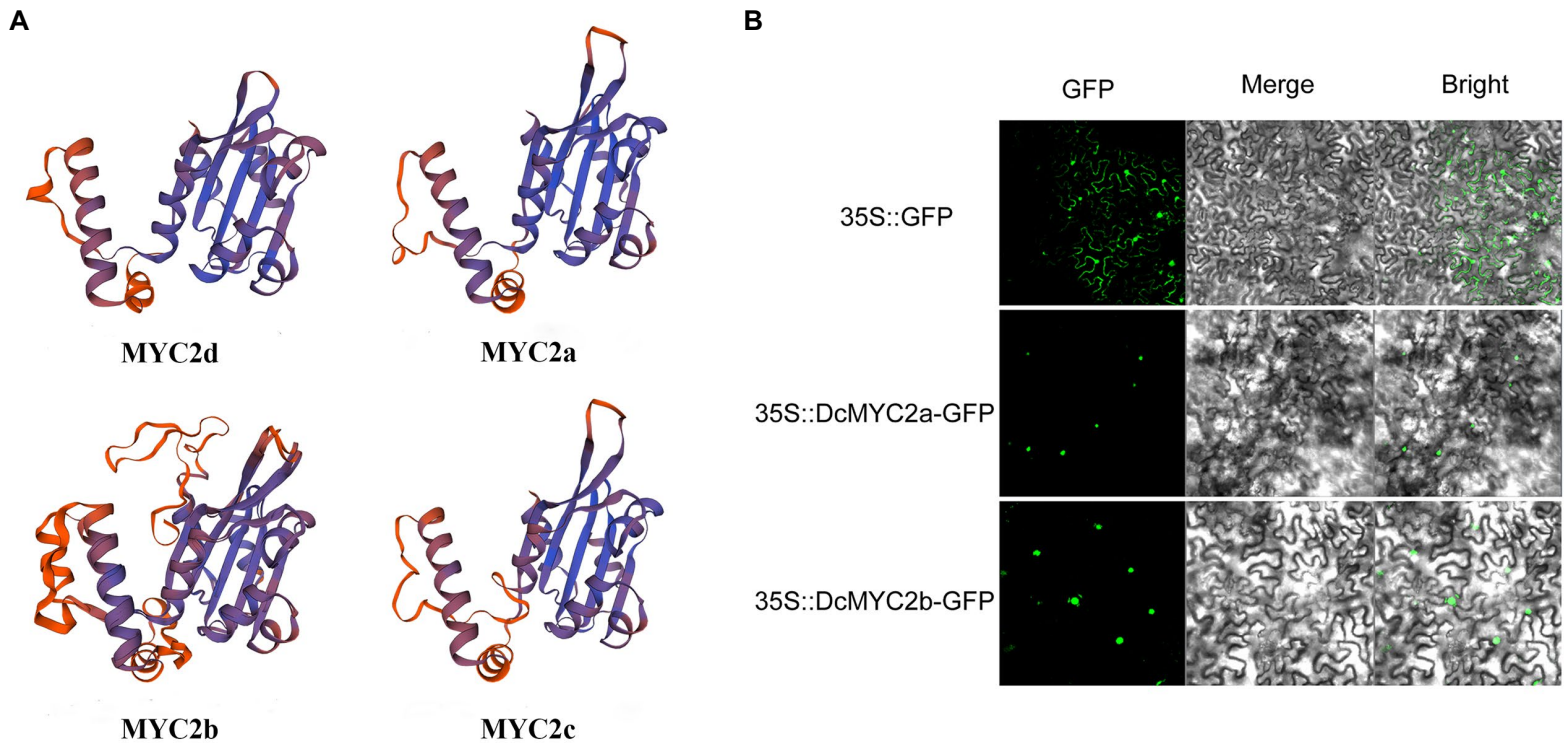
Prediction model of protein tertiary structure of subgroup IIIe members and subcellular localization. **(A)** Protein tertiary structure of DcMYC2a/2b/2c/2d. **(B)** Subcellular localization of DcMYC2a/2b in tobacco cells.

The infection of P1 and exogenous JA significantly changed the expression of *D. catenatum bHLH* IIIe subgroup members. In order to better predict their functions, we predicted the protein secondary and tertiary structures of the DcbHLH IIIe subgroup members DcbHLH025(MYC2d)/026(MYC2a)/027(MYC2b)/028(MYC2c) (Table 2; Figure 6A). The protein secondary structure and tertiary structure analyses showed that DcMYC2a/2b/2c/2d had similar secondary structures, contained a large number of irregular curls and alpha helices, and each member contained a highly similar three-dimensional structure, which indicated that the members of this subgroup are functionally similar or redundant. Subcellular localization of DcMYC2a/2b was examined with the tobacco leaf transient expression system. Both proteins were able to detect GFP fluorescence signals in the nuclei of *N. tabacum* epidermal cells (Figure 6B), demonstrating DcMYC2a/2b encoded nuclear proteins.

**Table 2:** Prediction of protein secondary structure of subgroup IIIe members.

Gene ID	Rename	Clade	Alpha helix	Extended strand	Beta turn	Random coil
LOC110094435	DcbHLH025(MYC2d)	IIIe	42.86%	12.55%	3.90%	12.55%
LOC110116479	DcbHLH026(MYC2a)	IIIe	33.79%	12.44%	1.69%	52.07%
LOC110114462	DcbHLH027(MYC2b)	IIIe	33.96%	12.89%	2.04%	51.10%
LOC110092865	DcbHLH028(MYC2c)	IIIe	33.54%	11.13%	1.98%	53.35%

### Overexpression of *DcMYC2b* improved P1 tolerance in yransgenic Arabidopsis

**Figure 7:**
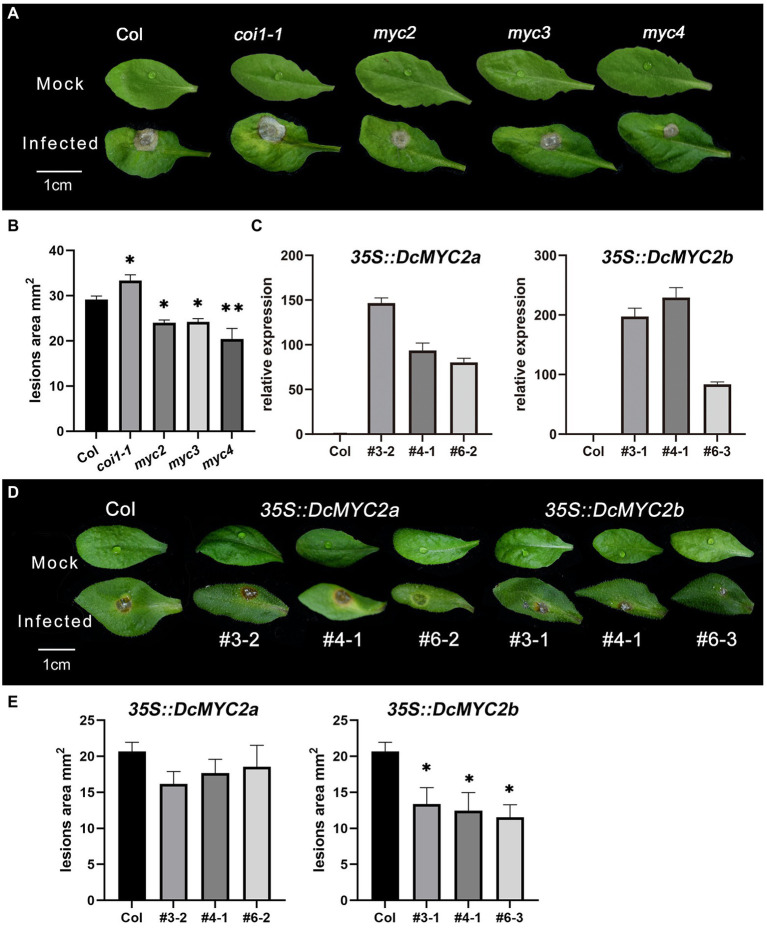
Functional validation of *DcMYCs* and *AtMYCs* genes. **(A)** Disease symptoms of leaves of Arabidopsis *myc2*, *myc3*, and *myc4* mutants following inoculation with *Sclerotium delphinii* at 48 hpi. Bar = 1 cm. **(B)** Lesion size of leaves of Arabidopsis *myc2*, *myc3*, and *myc4* mutants at 48 hpi. Significant difference (compared with the Col, ^*^p <  0.05, ^**^p <  0.01). **(C)** Relative expression of *DcMYCs* in Arabidopsis *DcMYCs*-OE strain. The abscissa represents different lines of *DcMYCs*-OE. **(D)** Disease symptoms of leaves of Arabidopsis *DcMYC2a/2b*-OE at 48 hpi. Bar = 1 cm. The # represents different lines. **(E)** Lesion size of leaves of Arabidopsis *DcMYC2a/2b*-OE at 48 hpi. The ^*^ shows a significant difference at *p* <  0.05 compared with the Col.

As Arabidopsis is also the host of P1, in order to further explore the function of MYC TFs, we obtained Arabidopsis mutants *myc2/3/4* and *DcMYC2a/b*-OE plants of Arabidopsis and infected their isolated leaves with P1. The results showed that Arabidopsis mutants *myc2/myc3/myc4* leaves had significantly smaller lesion size (24.01/24.21/20.42 mm^2^) than Col-0 (29.15 mm^2^) after P1 infection, while the leaves of *coi1-1* displayed bigger lesion size (33.33 mm^2^, Figures 7A,B; Supplementary Table S11), suggesting that *AtMYC2/3/4* function in Arabidopsis as negative regulators of downstream genes of JA signaling pathway related to P1 defense, which is consistent with the result of inoculation assays of necrotrophic pathogen *B. cinerea* in Arabidopsis *myc2* mutant (Song et al., 2014). Three independent homozygous lines of *DcMYC2a/b* were selected (*OE3/4/6* for *DcMYC2a* and *OE3/4/6 DcMYC2b*) with high gene expression levels in the T3 generation. *DcMYC2a* and *DcMYC2b* have higher expression than Col-0 in their respective *DcMYC2a-*OE and *DcMYC2b*-OE lines (Figure 7C), indicating that *DcMYC2a-*OE and *DcMYC2b*-OE lines were successfully constructed. After infection with P1, the lesion size of *DcMYC2a*-OE lines (18.31 mm^2^) was not significantly different from that of Col-0 (20.67 mm^2^), while the lesion size of *DcMYC2b-*OE lines (15.30 mm^2^) was significantly smaller than that of Col-0 (Figures 7D,E; Supplementary Table S12), suggesting that *DcMYC2b* may be a positive regulator in the JA-mediated resistance of *D. catenatum* to P1.

## Discussion

*Dendrobium catenatum* is an important economical medicinal plant under the forest. The quality of near-wild cultivation is significantly better than that of facility cultivation (Chen et al., 2020b). However, *D. catenatum* cultivated in the near wild is vulnerable to harsh environmental conditions, most prominently southern blight disease (Chen et al., 2019a). Searching for effective strategies to improve the resistance of *D. catenatum* under near-wild conditions becomes a difficult problem. The members of the family bHLH TFs play vital roles in plant growth, development, light signal transduction, and stress responses (Abe et al., 2003; Castelain et al., 2012; Liu et al., 2013; Yao et al., 2018; Zhang et al., 2020b). They are also involved in the crosstalk of hormone signaling, including abscisic acid (ABA), JA, brassinosteroid (BR), salicylic acid (SA), and ethylene (ET) (Murre et al., 1989; Heim et al., 2003; Simionato et al., 2007; Feller et al., 2011), and they are pivotal for the plant growth and survival in the environment. In our study, we constructed an evolutionary tree by combining DcbHLH with AtbHLH and OsbHLH, which are taxonomically well-defined, as well as bHLH (PebHLH and AsbHLH) of two *D. catenatum* relatives. A total of 108 *DcbHLH* genes were identified and divided into 12 subfamilies and 23 subgroups according to the phylogenetic analysis. However, DcbHLH was not identified in subgroup IIIa. We speculated that *bHLH* genes of subgroup IIIa may not be necessary to maintain the normal growth and development of *D. catenatum*, and it was lost in the evolution of *D. catenatum*.

An important regulation pathway is that transcription factors interact with cis-acting elements to express the genes involved in stress response (Mao et al., 2017) and developmental processes specifically (Li et al., 2012; Chen et al., 2018). Although functional studies of *bHLH* genes are still lacking in *D. catenatum*, the gene function can be predicted by the upstream cis-acting elements and the expression pattern of these genes, and the published *bHLH* gene functions of other species. The cis-acting element analysis pointed out that the *DcbHLH* genes contain a large number of cis-acting elements related to plant growth and development, and response to hormones and external stress, which indicated the importance of *bHLH* genes in plant growth and development as well as response to stress. For example, overwhelmingly members of subfamily VII belong to the PIF family (Paik et al., 2017). In Arabidopsis, PIFs interact with phytochrome (PFR) and crosstalk with other signaling pathways to regulate plant growth and response to environmental signals, including light and stress (Castillon et al., 2007). In our study, members of the bHLH VII subfamily of *D. catenatum* contain a large number of light-responsive elements, suggesting the potential functions of the members of subfamily VII in responding to light signals and participating in growth and defense mechanisms. Arabidopsis bHLH IIIe TF MYC5 has redundant functions with MYC2, MYC3, and MYC4, which interact with MYB21 and MYB24 to form the bHLH–MYB complex to regulate stamen development (Qi et al., 2015). The homologous genes of *AtMYC2/3/4/5* and *DcMYC2a/2b/2c* show high expression levels in pollens except for *DcMYC2d*, which illustrated that DcbHLH IIIe TFs may affect the pollen development of *D. catenatum.* Besides, *AtMYC5* is a positive regulator of signal transduction under salt stress (Ramsay and Glover, 2005). We found that *DcMYC2a* and *DcMYC2c* were strongly induced by salt stress, which may have redundant functions in regulating plants to adapt to high salt environments. In Arabidopsis, *ICE1* encodes an MYC-type bHLH TF that directly binds the promoter of the *C-Repeat Binding Factor/Dehydration-Responsive-Element-Binding protein* (*CBF/DREB1*) protein-encoding gene and activates its transcription to improve cold tolerance through an ABA-independent pathway (Stockinger et al., 1997). *DcbHLH017* is highly homologous with *AtICE1* and is highly expressed in plants under cold stress. We speculated that it may play an important role in *D. catenatum* response to cold stress.

The phytohormone JA plays a vital role in plant development and the response to various stresses. Exogenous JA could enhance the resistance of *D. catenatum* to P1 and delay the disease time. Phe, as an inhibitor of LOX activity and a key gene in the JA synthesis pathway, significantly reduced the resistance to P1 and inhibited the expression of *DcPR3* and *DcLOX2*, which are marker genes for JA-regulated pathogen responses. In addition, the expression of *DcLOX2* in the P1 + JA sample was significantly higher than in the P1 sample. These results demonstrated the importance of the JA signaling pathway in the resistance of *D. catenatum* to P1 infection. The MYC2 TFs are core regulators of the JA signaling pathway (Kazan and Manners, 2013). In previous studies, the resistance of *MYC2* to a necrotrophic pathogen exhibited species specificity (Song et al., 2014; Du et al., 2017). In Arabidopsis, the *MYC2* plays a negative regulatory role in plant resistance to the necrotrophic pathogen. Consistently, *myc2* mutants are more resistant to the necrotrophic pathogen, such as *Botrytis cinerea*, than the wild type, due to the fact that the *MYC2* interacts with *EIN3* and inhibits the function of *EIN3*, thereby inhibiting ethylene-mediated plant resistance to a pathogen (Song et al., 2014). Our experiments also proved that the Arabidopsis *myc2* mutant exhibited a bigger lesion size than Col-0 when infected with P1. Arabidopsis *MYC3* and *MYC4* are homologous genes to *MYC2*, and they have redundant functions and mediate a variety of physiological processes regulated by JA (Kazan and Manners, 2013; Schweizer et al., 2013; Zhang et al., 2020a). In our study, the phenotype of Arabidopsis *myc3* and *myc4* mutants infected by P1 was consistent with *myc2* mutants, indicating that they had certain similarities in function. When one MYC gene loses its function, other *myc* genes cannot completely replace its function, indicating that they had unique characteristics in JA signaling pathway. However, in *Solanum lycopersicum*, *SlMYC2* positively regulates both wound and pathogen response genes, but it cannot change the increased disease resistance of the Arabidopsis *myc2-2* mutant, and thus, *SlMYC2* and its Arabidopsis homologs have different action modes in regulating pathogen response genes (Du et al., 2017). The functional differences between AtMYC2 and SlMYC2 TFs may not be due to their different binding capacities, but rather to their different transcriptional regulatory activities, as *SlMYC2* and *AtMYC2* contain a highly conserved basic region that mediates binding to G-boxes of their target gene promoters (Toledo-Ortiz et al., 2003; Kazan and Manners, 2013). *SlMYC2* and *AtMYC2* may recruit different partner proteins while regulating pathogen-regulated gene transcription. *DcMYC2a/2b/2c/2d* had close homology with *AtMYC2/3/4/5*, and the expression of *DcMYC2a/2b/2c/2d* was significantly changed after the plants were treated with exogenous JA and infected with P1. However, the expression patterns of these four duplicated *DcMYC2* genes showed significant differences. *DcMYC2a* exhibited negative regulation under both JA and P1, but there was no significant difference in the lesion size between the *DcMYC2a*-OE and wild-type (Col-0) plants, possibly due to the fact that DcMYC2a was involved in other JA-mediated pathways. Instead, the *DcMYC2b* expression pattern suggested that it may be a positive regulator of the JA-related defense pathway against pathogens. It was confirmed by P1 inoculation assays of Arabidopsis *DcMYC2b*-OE plants, whose lesion size was significantly smaller than Col-0. *DcMYC2b* and *AtMYC2* have opposite regulatory effects in the process of plant response to P1, which may be caused by their recruitment of different chaperones through some subtle differences between the secondary and tertiary structures of proteins. The expression patterns of *DcMYC2c* and *DcMYC2d* were upregulated by JA, but repressed by P1, suggesting that these two genes might be involved in JA-regulated growth and development pathways. We performed secondary and tertiary structure predictions of the above four proteins and found similar structures, but there were some differences. It is possible that these differences enable them to recruit different partner proteins in order to perform different functions in the JA signaling pathway. As the key step in JA signaling pathway, the mechanism by which *MYC2* genes and their downstream genes form a transcriptional regulatory network that affects disease resistance in plants remains to be further studied.

## Data availability statement

The original contributions presented in the study are included in the article/Supplementary material, further inquiries can be directed to the corresponding authors.

## Author contributions

CL: conceptualization, experimentation, writing of the original draft, reviewing, and editing. XiC: conceptualization, experimentation, reviewing, and editing. QS: experimentation and data curation. XuC: data curation. MX and TY: experimentation. DS: data curation and editing. LW and DC: writing, reviewing, and editing. ZH and JS: conceptualization, writing, reviewing, and editing. All authors contributed to the article and approved the submitted version.

## Funding

This work was supported by the National Natural Science Foundation of China (31901235), the Major Science and Technology Projects of Yunnan Province (202102AE090042), and the Scientific R&D Foundation for Talent Start-up Project of Zhejiang A&F University (2019FR010).

## Conflict of interest

The authors declare that the research was conducted in the absence of any commercial or financial relationships that could be construed as a potential conflict of interest.

## Publisher’s note

All claims expressed in this article are solely those of the authors and do not necessarily represent those of their affiliated organizations, or those of the publisher, the editors and the reviewers. Any product that may be evaluated in this article, or claim that may be made by its manufacturer, is not guaranteed or endorsed by the publisher.

## Footnotes

^1^ https://www.arashare.cn/index/Product/index.html

^2^ https://www.ncbi.nlm.nih.gov/genome/

^3^ http://pfam.Xfam.org/

^4^ http://pfam.xfam.org/

^5^ http://smart.emblheidelberg.de/

^6^ https://web.expasy.org/protparam/

^7^ https://evolgenius.info/evolview-v2/#login

^8^ http://gsds.cbi.pku.edu.cn/

^9^ http://meme-suite.org/tools/meme

^10^ http://bioinformatics.psb.ugent.be/webtools/plantcare/html/

^11^ https://imagej.nih.gov/ij/download.html?utm_source=ld246.com

^12^ https://npsa-prabi.ibcp.fr/cgi-bin/npsa_automat.pl?page=npsa_sopma.html

^13^ https://swissmodel.expasy.org/

^14^ https://saves.mbi.ucla.edu/

## Supplementary material

Supplementary Figure S1 Cis-acting elements in the 1,500 bp promoter regions of the *DcbHLH* genes. Different color boxes represent different clades. Solid boxes of different colors represent different cis-acting elements, and the legend is on the right side of the figure.
