# Supplementary figures and images for: Genome-wide analysis of basic helix–loop–helix genes in Dendrobium catenatum and functional characterization of DcMYC2 in jasmonate-mediated immunity to Sclerotium delphinii
Source: Front Plant Sci. 2022 Aug 2;13:956210. doi: 10.3389/fpls.2022.956210 (PMC9378844; doi:10.3389/fpls.2022.956210)

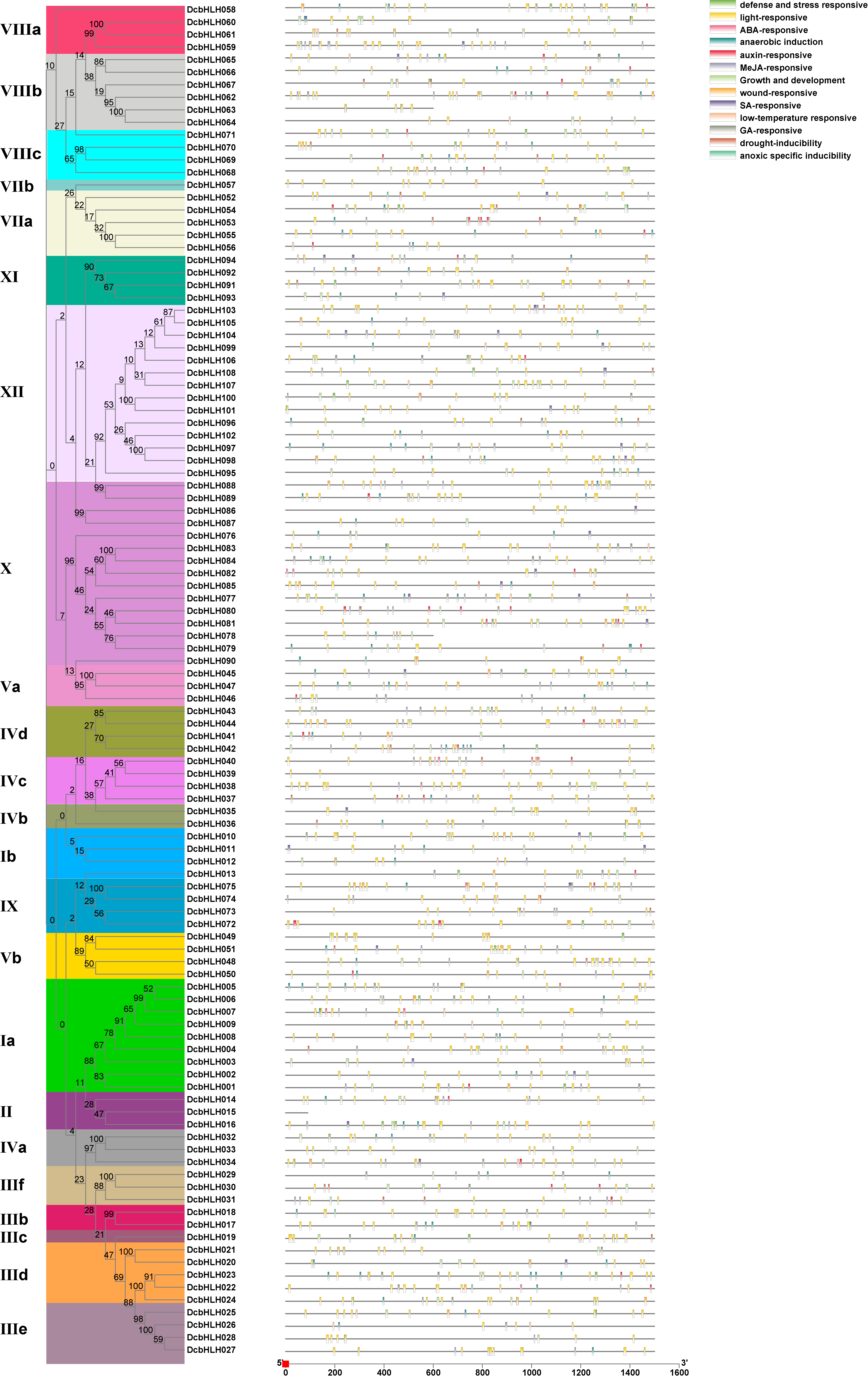

Supplement: Supplementary Figure S1 — Cis-acting elements in the 1,500 bp promoter regions of the DcbHLH genes. Different color boxes represent different clades. Solid boxes of different colors represent different cis-acting elements, and the legend is on the right side of the figure. [file Image_1.TIF]
